# Use of Transcorneal Iris Photocoagulation to Facilitate Sector Iridectomy of Pigmented Iridal Tumors: A Case Series of Five Eyes (Three Dogs and One Cat)—Clinical Findings, Surgical Technique, Complications, and Outcome

**DOI:** 10.1111/vop.70076

**Published:** 2025-09-11

**Authors:** A. K. Shukla, P. Grest, N. Holz, A. Rampazzo, S. A. Pot

**Affiliations:** ^1^ Ophthalmology Section, Equine Department, Vetsuisse Faculty University of Zurich Zurich Switzerland; ^2^ Institute of Veterinary Pathology, Vetsuisse Faculty, University of Zurich Zurich Switzerland

**Keywords:** complications, demarcation, diode laser, iris melanocytoma, neoplasia, surgical excision

## Abstract

**Objective:**

To describe the use of transcorneal iris photocoagulation (TCIP) to improve intraocular visualization of intended incision lines, reduce hemorrhage, and facilitate excision of pigmented iridal tumors in four canine eyes and one feline eye.

**Materials and Methods:**

A Rhodesian Ridgeback (treated bilaterally), a German Shepherd, a Labrador Retriever, and a Scottish Fold underwent sector iridectomy due to rapidly growing, pigmented, raised, iridal tumors affecting 1/4–1/3 of the iris circumference (3‐ to 4‐clock hours). A diode laser was used to delineate the intended sector iridectomy incision lines, approximately 1–2 mm away from the grossly visible tumor margins, with the aim of improving visualization to achieve tumor‐free margins and reduce tissue handling/trauma.

**Results:**

All iridal tumors were removed en bloc with tumor‐free margins on histopathology. The most common histologic diagnosis was iris melanocytoma (3/5 eyes). The most common intra‐ and postoperative complications included hyphema and fibrin clot formation (5/5), posterior synechia formation of iris wound margins (5/5 eyes), and photophobia (3/5 eyes). Two eyes required intracameral tissue plasminogen activator (tPA) injections within 2 weeks of surgery. The follow‐up period for all operated eyes ranged from 5 to 14 months. Vision was retained in all eyes, with no grossly apparent tumor regrowth within the follow‐up times included for each case in the present series.

**Conclusions:**

The use of TCIP to delineate intended incision lines improved intraocular visualization of sector iridectomy surgical margins in this case series. While hyphema and fibrin clot formation still occurred, they were successfully managed.

## 1 Introduction

Melanocytic neoplasia is considered the most common anterior uveal neoplasm in both canine and feline patients [1, 2]. In canine patients, both melanomas and melanocytomas have been reported, and both may present as raised, solitary masses apparent on the anterior surface of the iris or ciliary body [1, 2, 3]. Single masses are reported in feline patients; however, cats display a propensity to develop a more diffuse expression of pigmented iridal neoplasia [1, 2, 4, 5]. It is widely accepted that the risk of metastatic disease is higher in felines versus canines, though the visible appearance of the lesion is not necessarily indicative of the biological behavior in either species [2, 3, 5, 6]. These tumors present a challenge for the veterinary ophthalmologist and a difficult decision for the pet owner, as the desire to retain visual, non‐painful eyes must be balanced with the potential risk such intraocular neoplasms may present to the patient [5, 7].

Treatment strategies to address pigmented iridal tumors include therapeutic transcorneal iris photocoagulation (TCIP), sector iridectomy, and enucleation [1, 4, 6, 8, 9]. For the purpose of clarity, the authors of the present study use “therapeutic TCIP” to refer to the use of a diode laser to treat the tumor itself, and “TCIP” alone for the use of the diode laser to delineate surgical incision lines on grossly unaffected iridal tissue around a lesion. Therapeutic TCIP is an alternative therapy to enucleation in certain cases; however, multiple treatments may be required depending on the thickness of the tumor or episodes of recurrence, and the spread of tumor cells secondary to treatment may be a concern [4]. In cases where a solitary, well‐defined, raised tumor is noted, sector iridectomy may present an effective treatment strategy instead of therapeutic TCIP, allowing for retention of the globe and vision [1, 8, 9]. A recent study describing sector iridectomy in 13 canine patients used an ab interno and ab externo approach aided by a Fugo plasma blade (FPB) with good long‐term results [8]. However, a common complication included cataract development postoperatively, and the FPB is no longer commercially available [8]. Other reports detailing surgical excision of pigmented iridal tumors in small animal patients are limited [9, 10].

Removal of iridal tissue may be challenging due to the friable nature of the iris and its vascular supply [1]. Iridal incision may result in immediate hemorrhage, hampering visualization of surgical landmarks and proposed incision sites, and threatening effective en bloc excision of iridal tumors. The authors of the present study theorized that demarcation of the proposed iridal incision sites prior to entry into the anterior chamber (AC) could minimize bleeding and improve visibility, which might in turn support the surgical removal of the target tissue.

To the authors' knowledge, the delineation of proposed surgical margins of pigmented iridal tumors using diode laser TCIP to improve visualization during surgical resection has not been described. In this case series, the authors detail the sector iridectomy surgical techniques utilized to excise five pigmented iridal masses affecting three dogs (one patient treated bilaterally) and one cat, following delineation of normal appearing iridal tissue 1–2 mm away from visible tumor margins using TCIP. The surgical outcomes and relative merits of this technique with regards to improving intraocular visibility and facilitating excision of the described tumors en bloc are discussed, as well as the complications encountered.

## 2 Materials and Methods

**FIGURE 1:**
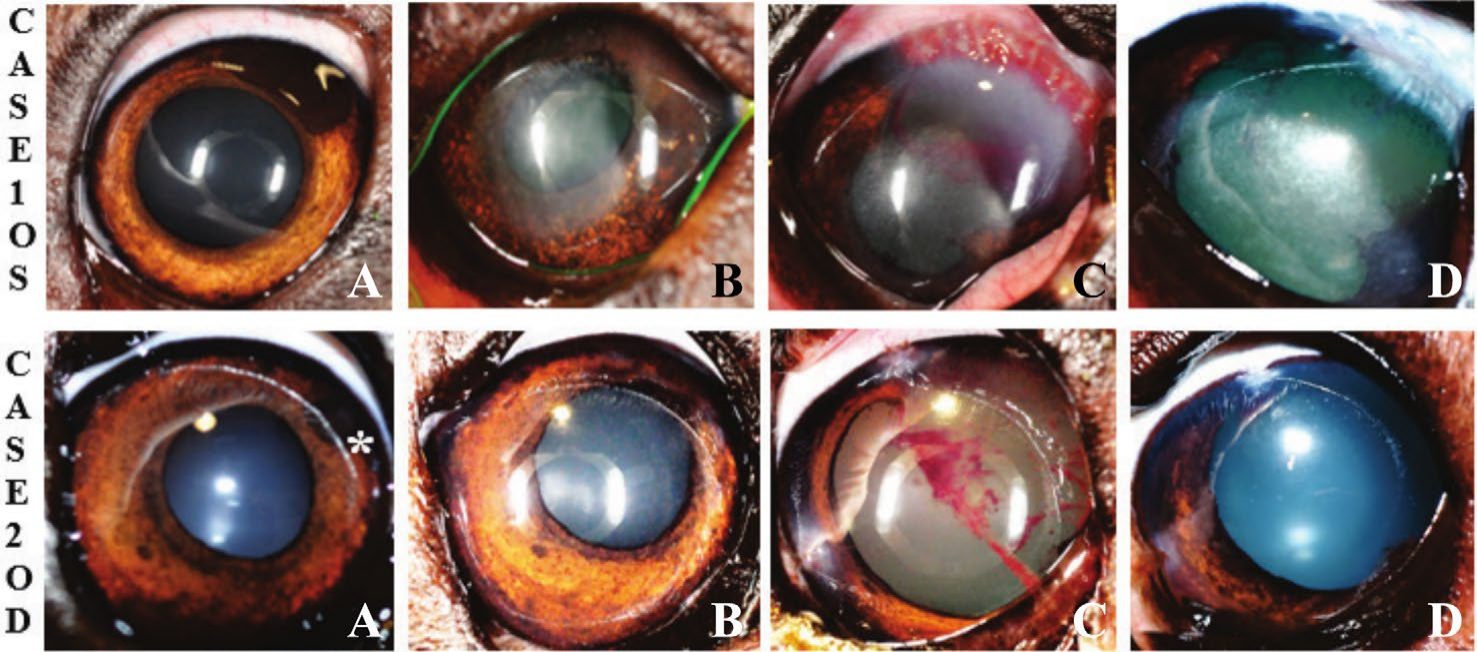
Representative images of canine Case 1, left eye and canine Case 2, right eye. (A) Appearance of iridal neoplasms at presentation. (B) Appearance of iridal neoplasms post therapeutic transcorneal iris photocoagulation (TCIP), displaying tumor progression and corneal opacity. (C) Postoperative appearance within 2 weeks of sector iridectomy. (D) Appearance at last follow up with no apparent tumor regrowth.

**FIGURE 2:**
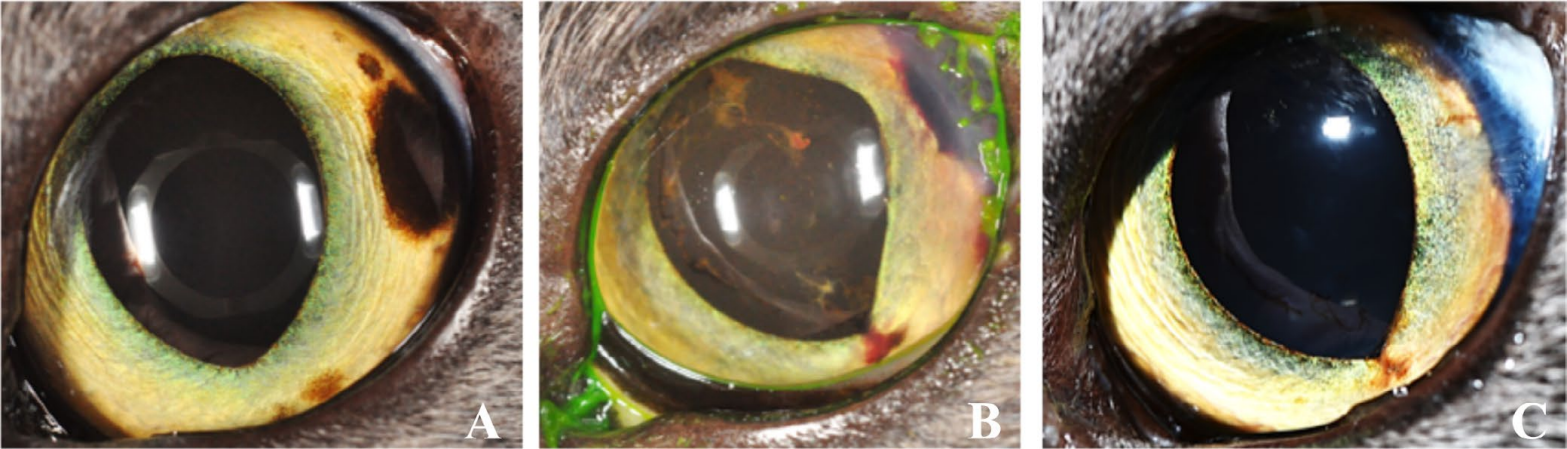
(A) Case 4 feline, left eye at presentation. (B) Nine days post sector iridectomy and therapeutic transcorneal iris photocoagulation (TCIP). (C) 1 year postoperatively with no grossly evident tumor regrowth.

The present study is a retrospective case series. All animals were presented to the Vetsuisse Faculty at the University of Zurich in 2023 and 2024 for further evaluation of an apparently rapidly growing pigmented, raised mass affecting a part, or parts, of the iris in one or both eyes. History, examination findings, and preoperative diagnostics for the individual cases are summarized in Table 1 and depicted in Figure 1 (canine cases 1–2) and Figure 2 (feline case). An ophthalmic examination was performed by a veterinary ophthalmology resident in training and board‐certified veterinary ophthalmologist, including menace response, dazzle, palpebral, and pupillary light reflexes (PLRs), Schirmer Tear Test (STT; Schirmer Tear Test Standardized Sterile Strips, MSD Animal Health, omitted in the single feline patient), intraocular pressure (IOP) testing using rebound tonometry (Tonovet Type TV01), slit‐lamp biomicroscopy (Kowa SL‐17), fluorescein staining (Fluo strips), and indirect ophthalmoscopy (mPack Unplugged, Heine Optotechnik; both 2.2D Pan Retinal and 20D Volk lenses). Unless otherwise noted, ocular reflexes, STT results, fluorescein stain, and IOP values were considered within normal limits. Informed consent was provided by all clients, and all clients signed treatment consent forms prior to therapy being administered.

**TABLE 1:** Signalment, history, pre‐operative diagnostics, therapy, and follow‐up for 5 eyes prior to and following sector iridectomy (SI).

Case	Signalment	History	Preoperative diagnostics	Therapy prior to SI	Timeline to SI	Therapy post‐SI	Diagnosis	Last follow‐up and CE findings
1	2.5‐year‐old MC RR	OS Rapidly growing iridal mass	Ocular US^a^: ICA and CB unaffected	3× therapeutic TCIP^b^, ^c^	6 months due to rapid mass growth despite therapeutic TCIP	Topical NSAID, antibiotic, lubricant, and systemic NSAID and antibiotic	Iris melanocytoma	11 months Visual, no regrowth, posterior synechia, corneal deposits, dyscoria, photophobia
		OD Pigmented lesion since puppyhood	Declined	4× therapeutic TCIP^b^, ^c^	8 months due to rapid mass growth despite therapeutic TCIP	As for OS	Iris melanocytic proliferation (hyperplasia or melanocytoma)	9 months Visual, no regrowth, posterior synechiae, corneal deposits, dyscoria, photophobia
2	6.5‐year‐old MC GS	OD Rapidly growing iridal mass, History of CSK OU	HRUS^d^: ICA unaffected Ocular US^a^: ICA and CB unaffected	1× therapeutic TCIP^c^, Tacrolimus eye drops BID	8 months due to rapid mass growth despite therapeutic TCIP	IC tPA injection 2 weeks post op Topical steroid, antibiotic, mydriatic, lubricant, and systemic steroid and analgesic	Iris melanocytoma	14 months Visual, no regrowth, posterior synechiae, dyscoria, corneal deposits, photophobia
3	6.5‐year‐old MI LR	OS Progressive increase in iridal pigment	Gonioscopy^e^: ICA unaffected HRUS^d^: ICA unaffected	None	1 month after presentation	IC tPA 6 days post op Topical NSAID, antibiotic, mydriatic, lubricant, and systemic steroid and antibiotic	Iris melanocytoma	5 months Visual, no regrowth, posterior synechiae, dyscoria
4	2‐year‐old MC SF cat	OS Rapidly increasing iridal pigment	Declined	None	6 weeks after presentation	Topical NSAID, antibiotic, mydriatic, lubricant, and systemic NSAID and antibiotic	Diffuse iris melanoma	12 months Visual, no regrowth, dyscoria

Abbreviations: BID, twice per day; CB, ciliary body; CE, clinical exam; CSK, chronic superficial keratitis; HRUS, high‐resolution ultrasound; GS, German Shepherd; IC, intracameral; ICA, iridocorneal angle; LR, Labrador Retriever; MC, male castrated; MI, male intact; NSAID, non‐steroidal anti‐inflammatory drug; OD, *oculus dexter*; OS, *oculus sinister*; OU, *oculus uterque*; RR, Rhodesian Ridgeback; SF, Scottish Fold; SI, sector iridectomy; TCIP, transcorneal iris photocoagulation; tPA, tissue plasminogen activator; US, ultrasound.

^a^ GE Vet, LOGIQ E10, Probe L8‐18i, GE HealthCare, General Electric Company.

^b^ DioVet Laser System 810 nm, IRIS Medical.

^c^ FOX A.R.C. Laser.

^d^ ellex eyecubed I3 40 MHz probe.

^e^ 19 mm gonioscopy lens, ocular instruments.

Surgery was performed on all patients under general anesthesia, following the administration of intravenous (IV) or intramuscular premedication, and intravenous induction, using combinations (drug and dose) of medications selected by the supervising anesthetist. All patients were maintained under isoflurane inhalant anesthesia (IsoFlo), with muscle relaxation achieved using 0.4 mg/kg rocuronium bromide IV (Esmeron) and additional 0.2 mg/kg doses as needed throughout anesthesia. Systemic antibiotics were administered at the discretion of the treating ophthalmologist.

Prior to TCIP, all eyes were prepared for ophthalmic surgery using a 2% povidone iodine flush (NaCl 0.9%, Fresenius Kabi, Betadine) and 2–3 drops of topical anesthesia (Oxybuprocaine 0.4%, Thea Pharma) applied afterwards. All surgeries were performed using a ceiling‐mounted surgical microscope (Leica Microsystems M844/M820 230V using 5–9× magnification) with a built‐in IRIS medical diode laser eye safety filter.

Transcorneal iris photocoagulation was performed prior to sector iridectomy using a diode laser (Fox A.R.C. Laser 810 nm, HS18004 handpiece) to demarcate the intended surgical incision sites, taking care to keep the beam of the laser focused, and using an operating microscope to allow direct visualization of the effect of the laser. A 200 mW power setting and continuous wave (CW) were used in all canine patients to create radial demarcation lines on healthy appearing iridal tissue from the pupillary margin to the ICA, 1–2 mm away from visibly abnormal tissue. The resultant radial incision demarcation lines made on either side of the tumor were approximately 0.2 mm wide. In the single feline patient, therapeutic TCIP was performed on the flat pigmented iridal lesions at the 4‐clock hour, and 5‐ to 6‐clock hours in a clockwise direction, using a diode laser (DioVet Laser System 810 nm, IRIS Medical). These lesions were targeted to effect over 2 cycles, with a power of 700 mW, duration 600 ms, repeat interval 50 ms, and 0.3 mm spot size, with the aim of seeing rapid tissue contraction and apparent reduction in lesion size. Following this, a different diode laser (Fox A.R.C. Laser 810 nm, HS18004 handpiece) was used to effect to demarcate the intended surgical incision sites around the raised temporodorsal tumor. In this patient, a squarely demarcated area was created encompassing the entire circumference of the tumor, 1–2 mm away from the tumor margins, parallel to the ICA and pupillary margin, using a power setting of 400 mW. This resulted in a readily visible line that was less obvious at the ICA. Following TCIP, all eyes were re‐prepared for intraocular surgery.

**FIGURE 3:**
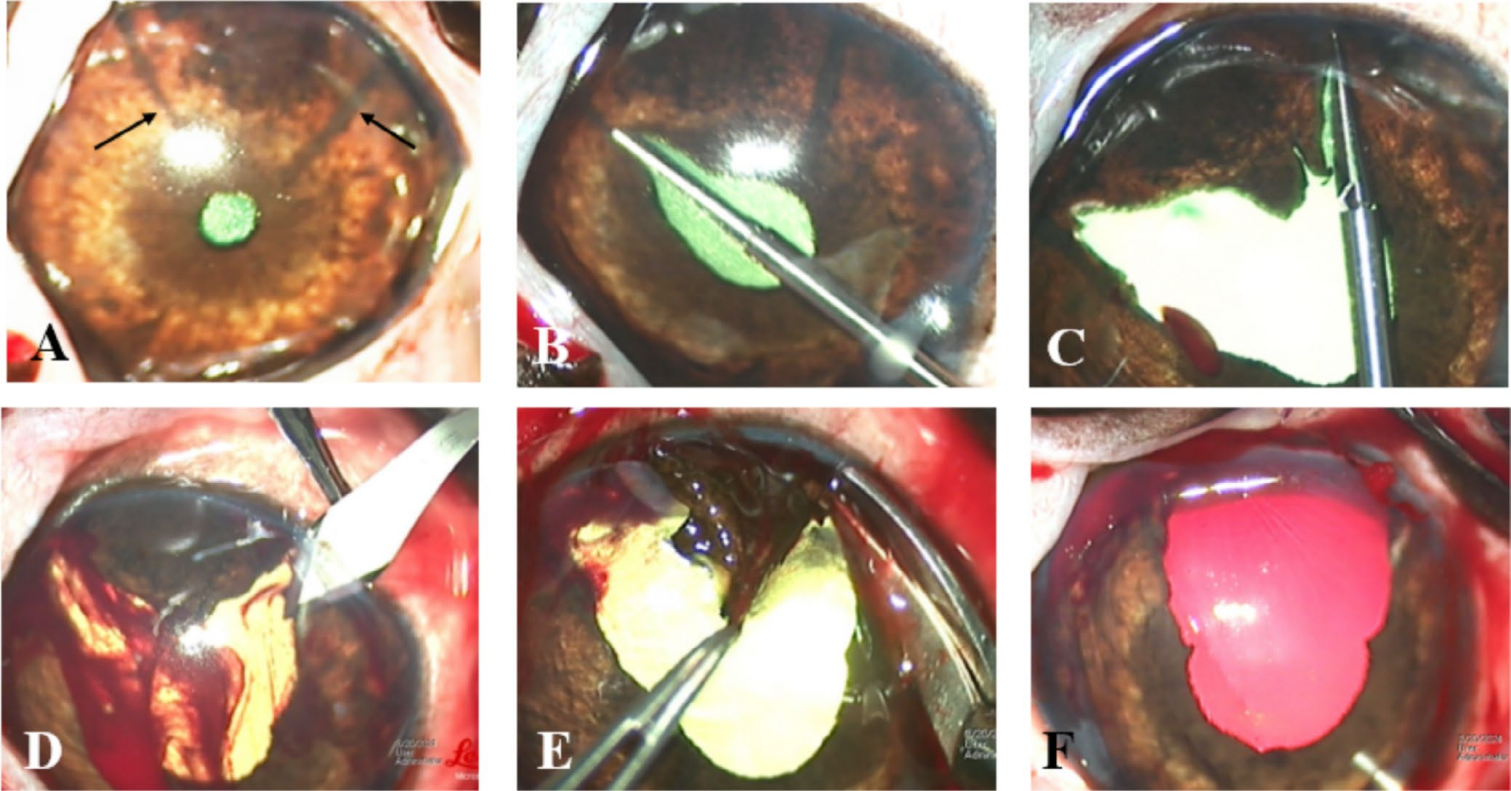
Intraoperative sector iridectomy Case 3, left eye. (A) Transcorneal iris photocoagulation (TCIP) was performed to delineate the intended excision margins (black arrows). (B) After a clear corneal stab incision and elevation of the iris with 1.8% viscoelastic, the iris is cut with scissors along the temporal TCIP demarcation line up to but not transecting the major arterial circle (MAC). (C) Incision along the nasal TCIP line toward the ICA using scissors and including the MAC at the iris base. (D) Moderate intraocular hemorrhage occurred from the second iris cut, as seen during the second corneal incision made from the 8 to the 5‐clock hours with a 2.8‐mm keratome and curved corneal scissors. (E) The iris tumor was extruded and excised en bloc at the iris base. (F) Appearance of the surgical site prior to corneal wound closure.

A 1 cm long lateral canthotomy was performed in all patients. Single or multiple, partial or full thickness, clear corneal incisions were made based on tumor location and surgeon preference, with entry into the AC achieved with a 2.8‐mm keratome (MSL28 slit‐angled 2.8‐mm Ophthalmic Knife, Mani Inc) and/or 19 g phaco lance (MVR‐Lance 19 g, Mani Inc); longer corneal incisions were completed using angled right or left corneal scissors (Figure 3). The AC was re‐inflated using viscoelastic according to surgeon preference (an‐bfh 1.8%, 2.2% an‐vision, and/or AJL Visco 3%). Sector iridectomy was then performed to resect all described tumors en bloc using fine iris scissors or intraocular scissors depending on tumor location. In the canine patients, radial demarcation lines were incised first, with the final incision extending along the iris base. In the feline patient, the initial incision was made parallel to the pupillary margin followed by the radial demarcation lines and completed at the level of the iris base. Intraocular hemostasis was achieved, as required, with the injection of intracameral adrenaline (Adrenalin Sintetica 1 mg/mL, Sintetica) and viscoelastic at the site of the hemorrhage to effect, resulting in effective tamponade. Following sector iridectomy, all corneal incisions were apposed using 9–0 polyglactin 910 (Ethicon Coated Vicryl) in a simple continuous or simple interrupted pattern with non‐buried knots according to surgeon preference, 1 mg (0.1 mL) of cefuroxime (ceFUROxime Labatec 750 mg reconstituted using NaCl 0.9%, Fresenius Kabi) was injected intracamerally, and the lateral canthotomy was routinely closed.

Following excision, all tissues were placed in microcassettes between 2 foam pads to prevent excessive rolling of the tissue specimens and then placed in 10% buffered formalin. Tissues were fixed for at least 24 h and routinely processed for histologic examination.

Initial postoperative re‐evaluation for all patients occurred within 2 weeks of surgery based on clinical progress (Figures 1 and 2). Additional postoperative re‐evaluation for all patients occurred within 4 weeks of surgery, with a general recommendation for 1–2 re‐evaluations per year for all patients (Figures 1 and 2). Postoperative therapy and adjustments to topical and systemic medications (determined at the discretion of the treating ophthalmologist) and postoperative diagnoses are summarized in Table 1.

## 3 Results

**FIGURE 4:**
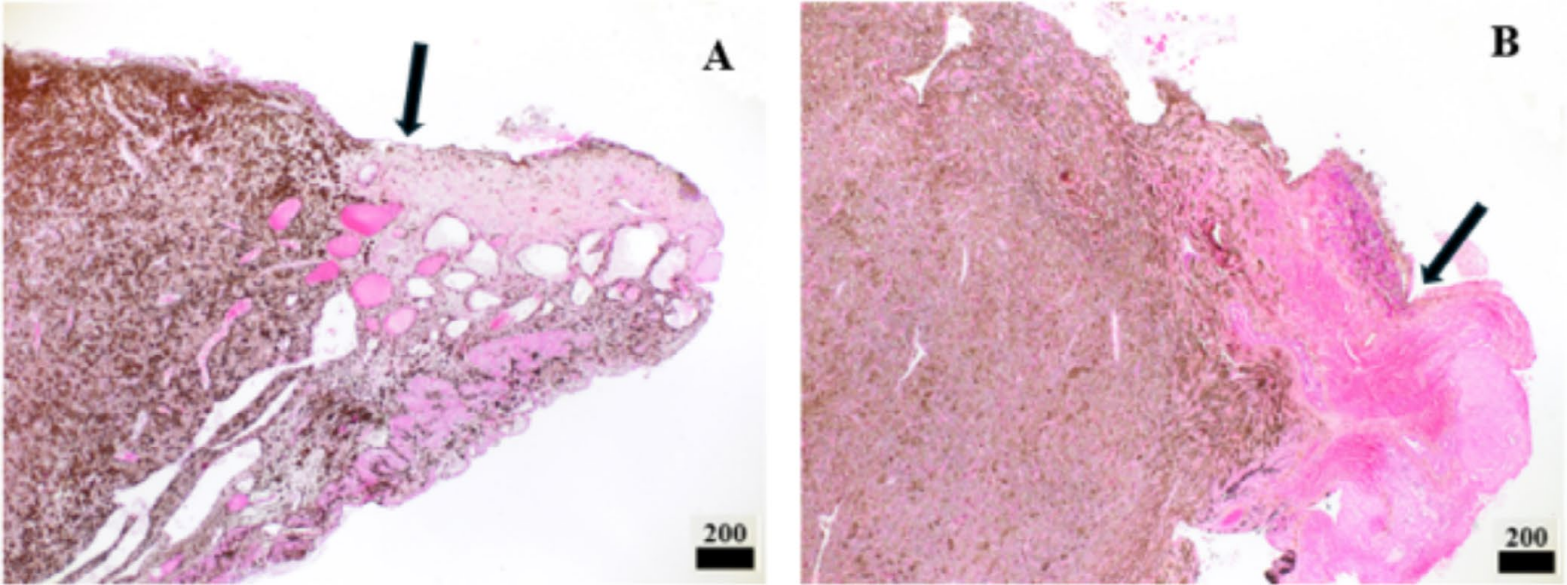
Representative photomicrographs of excised iridal masses from Case 1 (A), and Case 4 (B). A densely pigmented cell population with elevation of the iridal surface and a border of normal appearing iris (black arrows) is apparent, with the surgical margin beyond the normal appearing tissue (4× magnification, scale bar: 200 μm, hematoxylin and eosin stain).

Five eyes are included in this case series, all of which underwent TCIP to delineate the intended incision lines prior to sector iridectomy. One Rhodesian Ridgeback (Case 1, treated OU), 1 German Shepherd (Case 2, treated OD), 1 Labrador Retriever (Case 3, treated OS), and 1 Scottish Fold (Case 4, treated OS) are included (Table 1). Two patients (Case 1 OU, Case 2) underwent therapeutic TCIP prior to sector iridectomy (Table 2, Figure 1). The longest follow‐up period was 14 months (Case 2, OS), and the shortest follow‐up period was 5 months (Case 3). The size of the tumors that were excised ranged from approximately 1/4 to 1/3 of the iris circumference (Table 1). In all patients, a subjectively excellent tissue response, as determined by the rapid creation of a dark, thin, contracted line of tissue, was noted when the radial demarcation lines were made (Figure 3). Despite the use of TCIP, moderate intraocular hemorrhage was encountered in all patients upon incision of the iris base and major arterial circle (MAC) (Figure 3). All eyes displayed a variable degree of intraocular fibrin and anterior uveitis postoperatively, and all developed posterior synechiae between iris wound margins and anterior lens capsule with maintenance of a normal IOP at the last follow‐up examination. Two of the five eyes required injection of intracameral tPA within 2 weeks of surgery due to ongoing or worsening fibrin/blood clot formation. Focal anterior lens capsular fibrosis was noted in 1/5 eyes (Case 3), and 3/5 eyes developed subepithelial corneal lipid deposits throughout the follow‐up period (Cases 1–2, Figure 1). Photophobia was reported by the owners of Cases 1 and 2 (3/5 eyes) persisting until the last follow‐up. At the last follow‐up examination, 5/5 eyes were visual and comfortable, and without apparent tumor recurrence (Figure 1, Figure 2). The most common histopathological diagnosis was melanocytoma (3/5 eyes), followed by melanocytic proliferation (1/5 eyes), and feline diffuse iris melanoma (1/5 eyes), with no mitotic figures found in 10 high‐power fields in any of the cases. Further details regarding histopathologic diagnoses are detailed in Table 1 and depicted in Figure 4.

**TABLE 2:** Cases 1 and 2 treatment details prior to sector iridectomy.

Case	Treatment	Diode laser utilized	Laser settings	Postoperative therapy	Regrowth	Timeline
1	Therapeutic TCIP OU 7 days post presentation	DioVet Laser System 810 nm, IRIS Medical	OS: 700 mW‐1000 mW 800 ms‐1500 ms Repeat interval 50 ms 0.3 mm spot size, 2 cycles^a^ OD: 700 mW 800 ms Repeat interval 50 ms 0.3 mm spot size, 2 cycles^a^	OU: Topical NSAID and lubricant Systemic NSAID	Yes OU	7 weeks post 1st therapeutic TCIP
	Therapeutic TCIP OU 7 weeks post 1st therapeutic TCIP	FOX A.R.C. Laser	OS: 400–900 mW, 2 cycles^a^ OD: 200 mW, 2 cycles^a^	OS: Topical steroid, mydriatic and lubricant OD: Topical steroid and lubricant Systemic NSAID	Yes OU	3 weeks post 2nd therapeutic TCIP
	Therapeutic TCIP OU 3 weeks post 2nd TCIP	FOX A.R.C. Laser	OS: 200 mW‐600 mW, 3 cycles^a^ OD: 300 mW, 2 cycles^a^	OU: Topical steroid and lubricant Systemic NSAID	Yes OU	12 weeks post 3rd therapeutic TCIP
	Therapeutic TCIP OD 3 months post 3rd TCIP	FOX A.R.C. Laser	200 mW‐300 mW, 3 cycles^a^	Topical NSAID and lubricant Systemic NSAID	Yes	6 weeks post 4th therapeutic TCIP
2	Therapeutic TCIP OD 3 months post presentation	DioVet Laser System 810 nm, IRIS Medical	400 mW‐1000 mW 500 ms‐2000 ms Repeat interval 50 ms 0.3 mm spot size, 3 cycles^a^		Yes	6 weeks post therapeutic TCIP

Abbreviations: CE, clinical examination; OD, *oculus dexter*; OS, *oculus sinister*; OU, *oculus uterque*; TCIP, transcorneal iris photocoagulation.

^a^ An acceptable tissue response was evidenced by appreciable, rapid contraction of tissue and apparent reduction in tumor size.

## 4 Discussion

The purpose of this case series was to describe the use of TCIP prior to sector iridectomy as a method to improve intraocular visualization of intended incision lines, thereby facilitating en bloc surgical resection of pigmented tumors, and to describe intra‐ and postoperative complications in the cases presented here. In general, challenges encountered during sector iridectomy include difficulty visualizing planned surgical margins due to hyphema and tissue distortion following iridal incision [1]. In the present case series, the laser demarcation lines mitigated poor visibility of intended incision lines due to tissue distortion secondary to incision(s) of the iris and/or injection of viscoelastics and/or adrenaline. Although tissue distortion of iridal tissue may also be observed following photocoagulation itself, the authors found that applying a focused laser beam and taking a 1–2 mm margin from the grossly visible tumor prevented any clinically significant tissue distortion from occurring, resulting in a visibly “clean” margin of tissue between tumor and demarcation line. It is also important to note that improved intraocular visualization of the planned incision lines might have had other benefits that deserve further study, such as reduced tissue handling and reduction in intraocular hemorrhage.

Hyphema was the most common immediate postoperative complication in a retrospective study evaluating surgical treatment of iridal tumors in human patients [11], and was noted in all 13 canine patients following sector iridectomy in a recent publication by Dufour et al. [8], and all five patients presented here. In this case series, although hyphema and fibrin formation associated with anterior uvea disruption were effectively managed medically with anti‐inflammatory medications and intracameral injections of tPA, further studies could investigate how to minimize or avoid these complications altogether.

A complication noted by Dufour et al. following sector iridectomy included focal cataract development in four eyes which underwent ab interno removal of affected iridal tissue with the use of the FPB [8]. The authors hypothesized that lenticular trauma induced by the FPB was possible, resulting in a change in their surgical technique [8]. A total of 8/13 cases developed incipient cataracts in the Dufour et al. case series [8], whereas cataract development as a complication was not noted in the present study, which may be due to the minimally invasive nature of TCIP and the improvement in visualization that the laser demarcation lines afforded.

The two canine patients that underwent therapeutic TCIP prior to sector iridectomy developed lipid keratopathy. While corneal damage caused by therapeutic TCIP treatment might have been related to the development of lipid keratopathy in these patients, other causes, including but not limited to possible genetic predisposition, pre‐existing corneal disease, and use of topical and systemic steroids, cannot be excluded. Lipid keratopathy has also been reported following surgical debulking and diode laser photocoagulation of limbal melanomas in dogs [12], and following lamellar keratectomy and cryotherapy of canine limbal melanomas [13]. However, as in the present study, the mechanism for the development of lipid keratopathy in these cases is also unknown [12, 13].

Longer term complications following partial iris resection in human patients include photophobia [11, 14, 15], a complication seen in 1/2 canine patients following postero‐anterior cyclo‐iridectomy [9], and in 2/4 patients presented in this case series. These two canine patients (Cases 1 and 2) had iridal tumors excised from a dorsal quadrant, whereas the remaining canine patient's tumor (Case 3) was located ventrally. It is possible that photophobia was more apparent in Cases 1 and 2 due to a lack of third eyelid coverage. In the single feline patient, the pupillary margin could be retained allowing for improved iridal and pupillary mobility. This may explain why photophobia was not observed, in contrast to the canine patients where the pupillary margin was partially resected resulting in partial cycloplegia.

Local excision of pigmented uveal neoplasia is performed in selected human patients, and results in good outcomes in postoperative parameters including vision, recurrence rates, and patient survival [11, 15, 16, 17]. Parameters to pursue surgical excision in human patients include, amongst others, documented rapid growth, tumor size and location, lack of tumor spread, normal IOPs, and patient preferences [11, 14, 15, 17]. If a similar approach is to be adopted in veterinary ophthalmology, special care should be taken to avoid extrapolating the behavior and prognosis of intraocular neoplasia from one species to another. This is particularly true when approaching canine and feline anterior uveal melanocytic tumors, as they exhibit notable differences in biological behavior. Feline melanocytic neoplasms often exhibit a diffuse growth pattern, occasionally with areas of local consolidation, rather than the discrete, solid masses more commonly observed in dogs [1, 2, 3, 5, 7]. Accordingly, local excision can be a useful diagnostic tool to differentiate benign pigmentation from a melanocytic neoplastic process [6]. However, caution is advised before considering surgical resection alone to be curative in this species, and setting realistic expectations with the owner prior to surgical intervention remains an important aspect of clinical management.

## 5 Conclusions

The use of TCIP prior to sector iridectomy to delineate intended surgical margins improved intraocular visualization in the cases presented here. Hyphema and fibrin clot formation still occurred, though this was routinely managed and did not appear to have an obvious impact on visual outcome. Further studies utilizing this technique could focus on the reduction of hemorrhage and determining if the technique reduces tissue handling and facilitates en bloc excision of tumors, as theorized by the authors.

## Author Contributions

**A. K. Shukla:** conceptualization, data curation, investigation, methodology, project administration, writing – original draft, writing – review and editing. **P. Grest:** investigation, methodology, writing – review and editing. **N. Holz:** project administration, writing – review and editing. **A. Rampazzo:** methodology, project administration, supervision, writing – review and editing. **S. A. Pot:** methodology, project administration, supervision, writing – review and editing.

## Ethics Statement

Informed consent was provided by all owners of the patients described in this article. Clients were informed of the potential for metastatic disease both pre‐ and postoperatively, the potential for excision to be incomplete with a recurrence of tumor growth, which could necessitate a subsequent surgical procedure. Screening for metastatic disease, including thoracic radiographs, abdominal ultrasound, bloodwork and/or full body CT prior to surgery, was recommended for all cases. Enucleation of all described globes was discussed and declined by all clients. Treatment consent forms were signed by all clients prior to surgical intervention being undertaken.

## Conflicts of Interest

The authors declare no conflicts of interest.

## Data Availability

Data sharing not applicable to this article as no datasets were generated or analyzed during the current study.
